# Robustness Through Simplicity: A Minimalist Gateway to Neurorobotic Flight

**DOI:** 10.3389/fnbot.2020.00016

**Published:** 2020-03-16

**Authors:** Simon D. Levy

**Affiliations:** Computer Science Department, Washington and Lee University, Lexington, VA, United States

**Keywords:** drones, miniature aerial vehicles, spiking neural network, PID control, flight simulator

## Abstract

In attempting to build neurorobotic systems based on flying animals, engineers have come to rely on existing firmware and simulation tools designed for miniature aerial vehicles (MAVs). Although they provide a valuable platform for the collection of data for Deep Learning and related AI approaches, such tools are deliberately designed to be general (supporting air, ground, and water vehicles) and feature-rich. The sheer amount of code required to support such broad capabilities can make it a daunting task to adapt these tools to building neurorobotic systems for flight. In this paper we present a complementary pair of simple, object-oriented software tools (multirotor flight-control firmware and simulation platform), each consisting of a core of a few thousand lines of C++ code, that we offer as a candidate solution to this challenge. By providing a minimalist application programming interface (API) for sensors and PID controllers, our software tools make it relatively painless for engineers to prototype neuromorphic approaches to MAV sensing and navigation. We conclude our discussion by presenting a simple PID controller we built using the popular Nengo neural simulator in conjunction with our flight-simulation platform.

## 1. Introduction

Beginning with J.J. Gibson's pioneering research on visual perception (Gibson, 1979), decades of research in behavioral neuroscience have shown the importance of robust, tightly-coupled perception/action cycles in supporting successful movement (predation, obstacle avoidance) in challenging environments. This is especially true for flying animals like birds and insects, whose survival depends on overcoming of a variety of forces in three-dimensional space; most obviously, gravity (Floreano et al., 2009).

In attempting to build neurorobotic systems based on flying animals, engineers have come to rely on existing firmware and simulation tools designed for miniature aerial vehicles (MAVs). Although they provide a valuable platform for quick entrée into the world of first-person-view (FPV) racing or aerial photography (firmware), and the collection of data for Deep Learning and related AI approaches (simulation), such tools are deliberately designed to be as feature-rich and general as possible, to appeal to the widest audience. The most popular software tools support air, ground, and water vehicles and provide a hierarchy of safety mechanisms for minimizing the likelihood of injury and property damage. Unsurprisingly, the sheer amount of code required to support such broad capabilities can make it a daunting task to adapt these tools to building neurorobotic systems for flight.

In the remainder of this paper we present a pair of simple, object-oriented software tools—*Hackflight* and *MulticopterSim*—each consisting of a core of a few thousand lines of C++ code, that we offer as a candidate solution to this challenge. These software tools are built on the popular Arduino microcontroller platform and the popular video game platform Unreal Engine 4. By providing a minimalist application programming interface (API) for sensors and PID controllers, these tools make it relatively painless for engineers to prototype neuromorphic approaches to MAV sensing and navigation.

## 2. Hackflight

Hackflight is an open-source toolkit for building multirotor flight-control firmware and software. The project began in 2015 as an attempt by the author to build a simple open-source flight-control firmware program for MAVs using the Arduino platform (Banzi and Shiloh, 2014). At that time, as well as today, there were two major firmware projects for MAVs: ArduPilot (ArduPilot Dev Team, 2019a) and Cleanflight (Cleanflight Team, 2019). ArduPilot focuses on sophisticated mission planning with waypoint navigation and other features, and runs mainly on the Pixhawk flight controller. Cleanflight and its derivatives (Betaflight, Raceflight) are popular with FPV racing enthusiasts, and run on a broad variety of flight-control boards designed for FPV racing. (A more recent Cleanflight derivative, iNav, adds features for navigation and for fixed-wing aircraft). Although both projects can trace their origin to the Arduino platform, they have long since switched to using their own non-Arduino hardware drivers for sensing and motor control. Both projects are supported by large development teams and have a code base of several hundred thousand lines (see Table 1). Hackflight, by contrast, uses approximately 4,500 lines^1^.

**Table 1:** Approximate size of flight-control firmware packages.

**Package**	**Lines of code**
Cleanflight	851,659
ArduPilot	283,316
Hackflight	4,445

How can Hackflight get away with using to or three orders of magnitude less code than the two most popular flight-control firmware packages? As discussed in the sections below, we attribute this difference to a few important design principles: (1) limitation to multirotor vehicles, not fixed-wing or ground vehicles; (2) targeting programmers instead of general users; (3) Arduino compatibility; (4) simple object-oriented API.

### 2.1. Features

Unlike ArduPilot, which supports a variety of vehicle types (multirotors, fixed-wing aircraft, ground vehicles, marine vehicles), Hackflight supports only multirotors. Cleanflight and its derivatives, while supporting mainly multirotors (and perhaps fixed-wing aircraft), offer a variety of configuration features and flight modes (PID controllers), allowing everyone from beginners to professional racing pilots to use them. Hackflight, by contrast, uses only a the bare minimum of PID controllers necessary for stable flight, allowing you to create your own PID controllers with relative ease (see section 2.4 below).

### 2.2. Audience

Although both ArduPilot and Cleanflight are open-source, their target users are mostly non-programmers. There is therefore a heavy focus in both projects on GUI-based configurator programs. Hackflight, by contrast, is targeted toward engineers and researchers comfortable with coding in C++. Adding a feature to Hackflight therefore requires significantly less code support, enabling rapid prototyping of new sensors, PID, controllers, etc.

### 2.3. Arduino Compatibility

**Figure 1:**
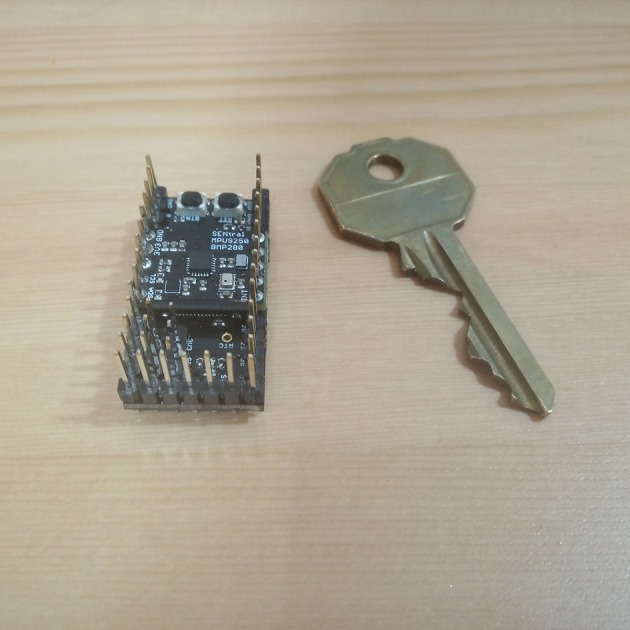
Arduino-compatible flight controller for Hackflight (total cost ~$55 U.S.).

As mentioned above, Hackflight began as the author's attempt to build a simple open-source flight-control program using the Arduino software libraries. Although Hackflight now supports a subset of the STM32F3/4 flight controllers supported by Cleanflight and its derivatives, our focus has always been on Arduino compatibility. Thanks to the recent availability of small, fast, 32-bit microcontroller development boards like Teensy and the STM32L4 line from Tlera Corporation^2^, Arduino compatibility is no longer tied to slower, eight-bit boards lacking floating-point support (see Figure 1). Arduino compatibility means that Hackflight can quickly exploit the increasing variety of new sensors available today, without the need to write a custom driver. Although the variety of neuromorphic sensors currently available cannot rival the variety of Arduino-compatible MEMS sensors (inertial measurement units, proximity sensors, and the like), we are optimistic that neuromorphic devices will follow the same trajectory; i.e., they will provide a UART or other low-level serial interface for working with Arduino and similar development platforms.

### 2.4. Simple Object-Oriented API

Hackflight is written entirely in C++, with the core components written in header-only style. Our focus is on object-oriented design, with most classes (altitude PID control, distance sensing) being subclasses of other, more abstract classes (PID controller, sensor). In addition to enabling extensive code re-use, this approach allows us to abstract the driver code for a component (sensor, motor) from the algorithms using that component (Madgwick quaternion filter, mixer). This clean separation allows Hackflight to be “dropped” directly into a simulation environment (through the use of C++ #include statements), without the need for “Hardware-In-the-Loop” (HIL), socket connections, or other indirect mechanisms (see section 3 below). Although both ArduPilot and Cleanflight separate the driver code from the algorithmic code, Hackflight's consistent use of object-oriented design allows us to avoid pre-processor macros (#ifdef … #else … #endif) that are used extensively in those two packages and can make it difficult to arrive at a basic understanding of much of the code.

**Figure 2:**
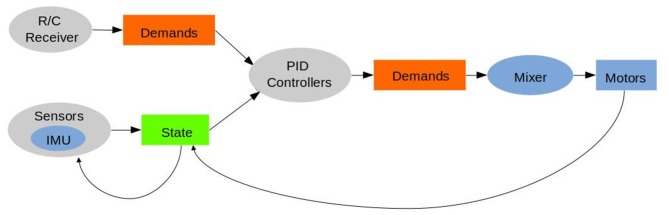
Hackflight main loop.

As well as keeping the codebase small, simple, and portable, these design principles support a more direct connection between the mathematical theory underlying flight control and its implementation in code. Figure 2 illustrates this point by showing the main loop in Hackflight. In the figure, each box (demands, state) represents a simple datatype in the C++ code, and each oval (R/C Receiver, Sensors, PID controllers, Mixer) represents an abstract class. Mathematically, then, each abstract class is a function from one datatype to another: Sensor:State ↦ State; PIDController:(State × Demands) ↦ Demands. We believe that this design principle makes Hackflight both easy to understand and simple to adapt.

**Figure 3:**
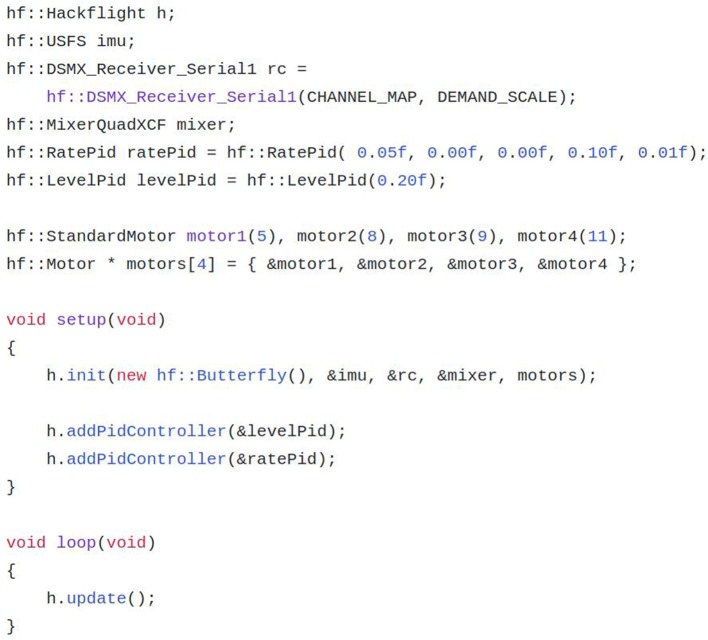
Sample Hackflight sketch for Arduino.

Figure 3 illustrates these principles by showing a complete Arduino firmware sketch (main program) for a quadcopter using the flight controller in Figure 1. As the sketch shows, Hackflight's simple API supports programs in which only the required components (microcontroller, IMU, receiver, PID controllers, mixer, motors) need to be specified (as opposed to choosing from a list of options with a control statement). This approach results in example programs that are easy for a programmer to read and to adapt for use with new sensors, vehicle designs and control paradigms.

## 3. MulticopterSim

Like Hackflight, MulticopterSim is designed as a minimalist solution to a difficult engineering problem; in this case, a physically realistic multirotor simulator general enough to interface with a variety of flight-control packages. As with similar efforts by others who have attempted to use a general-purpose robotics simulator like Gazebo (Koenig and Howard, 2004), our simulator began as a plugin for a more general robotics simulation platform, V-REP (Rohmer et al., 2013). The lack of realistic simulated camera images in these packages led us to a photo-realistic game engine, UnrealEngine4 (Sanders, 2016). Because UE4 is also used by Microsoft's popular AirSim (Shah et al., 2017) drone simulator, AirSim provides a useful frame-of-reference for MulticopterSim^3^.

In addition to its focus on Deep Learning, AirSim has since expanded to include support for self-driving cars, and provides Python APIs for remote operation of the vehicles. As with flight-control firmware discussed in the previous section, this rich set of features translates into significantly more code. Table 2 shows the relative sizes of AirSim and MulticopterSim, based on the same metric used in Table 1. As we saw with Hackflight, the design principles used in MulticopterSim help keep the codebase small, manageable, and easily extendable.

**Table 2:** Approximate sizes of two flight-simulation packages^*a*^.

**Package**	**Lines of code**
AirSim	77,600
MulticopterSim	2,266

a *The line count for MulticopterSim includes the module for Hackflight (see main text for details). For reference, the respective git commits were MulticopterSim: aec0ae8; AirSim: ca29068*.

The core of MulticopterSim is the abstract C++ *FlightManager* class. This class provides support for running the vehicle dynamics and the PID control regime (e.g., Hackflight) on its own thread, after it first disables the built-in physics in UE4. The dynamics we used are based directly on the model presented in Bouabdallah et al. (2004), written as a standalone, header-only C++ class that can be easily adapted for other simulators and applications if desired. This class also supports different frame configurations (quadcopter, hexacopter) via virtual methods. By running the FlightManager on its own thread, we are able to achieve arbitrarily fast updates of the dynamics and flight-control. We currently limit the update rate to 1kHz, based on the data output rate of current MEMS gyrometers. It would also be possible to run the dynamics and control on separate threads, though we have not yet found it advantageous to do that.

The FlightManager API contains a single virtual method, update(), which accepts the current time and the state of the vehicle (as computed by the dynamics), and returns the current motor values. The motor values are then passed to the dynamics object, which computes the new vehicle state. On the main thread, UE4's Tick() method queries the flight manager for the current vehicle pose (location, rotation) and displays the vehicle and its environment kinematically at the 60–120 Hz frame rate of the game engine. In a similar manner, the threaded *VideoManager* classes can be used to process the images collected by a simulated gimbal-mounted camera on the vehicle, using OpenCV (Bradski, 2000). An abstract C++ *Target* class supports modeling interaction with other moving objects having their own dynamics; for example, in a predator/prey scenario.

This simplicity of our flight-control scheme makes it easy to connect MulticopterSim to existing flight-control software like Hackflight, or to the Software-in-the-Loop (SITL) mechanism of ArduPilot (ArduPilot Dev Team, 2019b), as modules in the MulticopterSim codebase. With the Hackflight module, for example, we treat the control device (e.g., joystick, Xbox game controller) as “virtual receiver,” which provides the R/C Receiver signal shown at the top of Figure 2. Further, the abstraction provided by Hackflight for sensing and open-loop control allows rapid prototyping of hybrid control systems, as we describe in the next section.

## 4. Toward Neuromorphic Flight Control

**Figure 4:**
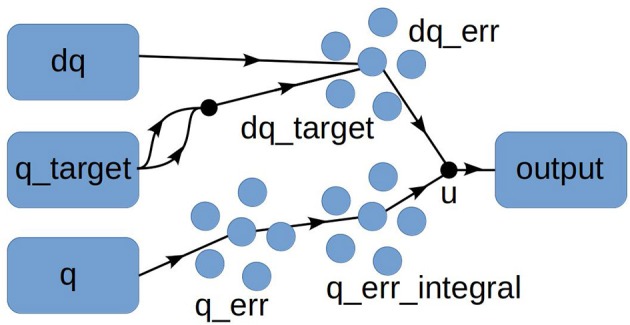
Nengo model for simple PID control.

As a demonstration of our approach, we used the Python-based Nengo neural simulator (Bekolay et al., 2014) to create a simple PID controller class for altitude hold. As shown in Figure 4, the controller consists of three populations of 200 spiking neurons: one population for computing the error between the target altitude and current altitude (*P* term); one for integrating the error (*I* term), and one for computing the error derivative *D*) term. (For this simple experiment we used only *P*.) The constants *K*_*P*_, *K*_*I*_, and *K*_*d*_ are implemented as arguments to the transform parameter of the nengo.Connection constructor; i.e., as connection weights between pools of neurons. We set the simulation time step to 0.001 s^4^ and used the default values for the remaining parameters in the Nengo class constructors. We made this Python class available to MulticopterSim by adding a UDP client/server module to MulticopterSim: the PID control code runs in Python as a server, and the C++ code for the simulator acts as a client for this server, sending the vehicle state to the server and getting back motor commands to fly the vehicle.

**Figure 5:**
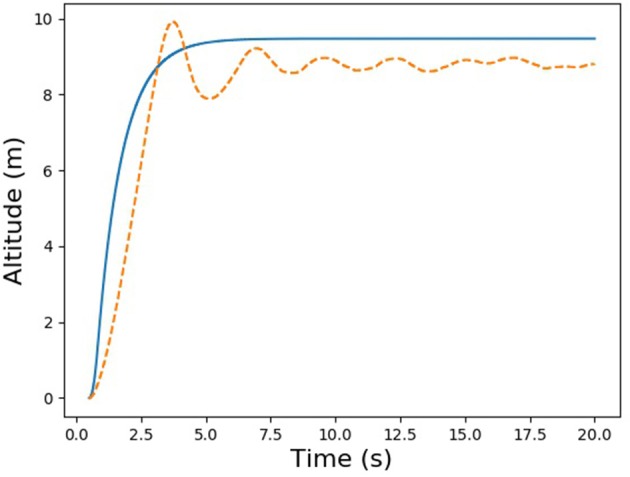
Comparison of traditional (solid line) and neural (dashed line) PID controllers.

For this trial experiment, we chose a simple PID control task common to flight-control systems like ArduPilot, namely, takeoff to a fixed altitude. We wrote two versions of the same basic Python server script. One version used the ArduPilot algorithm for altitude hold, with the error between the target and actual altitudes as a set-point for a secondary, velocity-based PID controller. The other version used the Nengo-based PID controller shown in Figure 4. Sample results for this experiments are provided in Figure 5. As the figure shows, the Nengo-based control compares favorably to the algorithm that computes the PID control signal in the traditional way, albeit with some oscillation and greater undershoot. Although this Nengo-based PID controller has been hand-tuned by us to work with our simulator, and could obviously use some improvement, it provides a simple proof of the feasibility of using an advanced neural simulator like Nengo with a real-time flight simulator, paving the way for more interesting experiments.

## 5. Conclusion and Future Work

As the closest robotic approximation to flying insects, birds, and mammals, miniature aerial vehicles (MAVs) offer a compelling new platform for research in neuromorphic sensing, notably in the realm of vision (Mitrokhin et al., 2019). Such research faces unique challenges.

In the physical realm, the current weight and form factor of commercially-available event-based dynamic vision sensor (DVS) devices makes them impractical for deployment on micro-scale aerial vehicles. We are currently experimenting with our recently-purchased DAVIS346 sensor H (40 × 60 × 25 mm, 100 g), using a RaspberryPi to convert the sensor's USB3 signal to UART (TTL) format for consumption by an Arduino-compatible microcontroller. If that arrangement proves successful, we will look into acquiring the much smaller mini-eDVS unit (18 × 18 × 7 mm, 3 g), from the same manufacturer^5^.

In simulation, the 60–120 Hz frame rate of game engines like UE4 and Unity (Menard, 2011) exceeds that of most commercially-available CMOS cameras but is inadequate for emulating the multi-kilohertz data rates enabled by DVS (Gallego et al., 2019). Hence, one of our current research directions involves modeling the DVS datastream directly from the dynamics of the vehicle and target object.

To extend our Nengo-based PID controller in a more biologically realistic direction, we are also experimenting with a Python version of our multirotor dynamics code, to exploit Nengo's support for reinforcement learning (Bekolay and Eliasmith, 2011). This paradigm would provide an accelerated way to develop neuromorphic flight controllers in an abstract mathematical simulation, to be validated by transferring them to MulitCopterSim, and eventually to an actual vehicle.

Finally, our Python-based client/server moduel will make it significantly easier to experiment with other neural simulators offering a Python API, including Brian (Stimberg et al., 2019) and NEURON (Hines and Carnevale, 2013).

For both real and simulated flying robots, we see our minimalist, integrated approach to software and firmware design as a promising direction for robust aerial neurorobotics.

## 6. Downloads

The software described in this paper can be downloaded from the following repositories:

- https://github.com/simondlevy/Hackflight
- https://github.com/simondlevy/MulticopterSim
- https://github.com/simondlevy/MulticopterSim/tree/NengoModule
- https://github.com/simondlevy/gym-copter.

## Data Availability Statement

All datasets generated for this study are included in the article/supplementary material.

## Author Contributions

The author confirms being the sole contributor of this work and has approved it for publication.

### Conflict of Interest

The author declares that the research was conducted in the absence of any commercial or financial relationships that could be construed as a potential conflict of interest.

## Footnotes

^1^To estimate the number of lines of code in each package, we cloned the package repository from github, ran the **cloc** program (https://github.com/AlDanial/cloc) in the root directory of the repository, and summed over the reported number of lines in C/C++ header files, C files, and C++ files. For reference, the respective git commits were: Hackflight: 206a6dd; Cleanflight: 83ed5df; Ardupilot: 87a5189.

^2^https://www.tindie.com/stores/TleraCorp/

^3^In March of 2017 the head of Microsoft's AirSim project contacted the author about using Hackflight as the flight-control software for AirSim, citing the design principles of Hackflight is the primary reason for this interest. After a licensing incompatibility ended up making this collaboration unfeasible, the author turned to developing quadcopter flight simulator from scratch, using UE4 and the Hackflight firmware.

^4^We chose this value as an order-of-magnitude approximation to the data output rate (DOR) of contemporary inertial measurements units. As one reviewer pointed out, it would also be useful to know how close to real-time such a model runs on the sort of standard CPU hardware that is available on a quadcopter (see future work section below).

^5^We thank a reviewer for suggesting the mini-eDVS, which was not available for purchase at the time of this writing.

## References

[B1] ArduPilot Dev Team (2019a). History of Ardupilot. Available online at: http://ardupilot.org/planner2/docs/common-history-of-ardupilot.html (accessed June 15, 2019).

[B2] ArduPilot Dev Team (2019b). Sitl Simulator (Software in the Loop). Available online at: http://ardupilot.org/dev/docs/sitl-simulator-software-in-the-loop.html (accessed June 17, 2019).

[B3] Banzi M. Shiloh M. (2014). Getting Started With Arduino. Sebastopol, CA: Maker Media.

[B4] Bekolay T. Bergstra J. Hunsberger E. DeWolf T. Stewart T. C. Rasmussen D.. (2014). Nengo: a python tool for building large-scale functional brain models. Front. Neuroinform. 7:48. 10.3389/fninf.2013.00048 24431999 PMC3880998

[B5] Bekolay T. Eliasmith C. (2011). A general error-modulated stdp learning rule applied to reinforcement learning in the basal ganglia, in Proceedings of the Conference on Computational Systems Neuroscience (COSYNE) (Salt Lake City, UT), 24–27.

[B6] Bouabdallah S. Murrieri P. Siegwart R. (2004). Design and control of an indoor micro quadrotor, in Proceedings of the 2004 IEEE International Conference on Robotics and Automation, ICRA 2004, April 26 - May 1, 2004 (New Orleans, LA), 4393–4398. 10.1109/ROBOT.2004.1302409

[B7] Bradski G. (2000). The OpenCV Library. Dr. Dobb's Journal of Software Tools.

[B8] Cleanflight Team (2019). Available online at: http://cleanflight.com (accessed June 15, 2019).

[B9] Floreano D. Zufferey J.-C. Srinivasan M. V. Ellington C. (2009). Flying Insects and Robots, 1st Edn. New York, NY: Springer Publishing Company, Incorporated 10.1007/978-3-540-89393-6

[B10] Gallego G. Delbrück T. Orchard G. Bartolozzi C. Taba B. Censi A. (2019). Event-based vision: a survey. CoRR, abs/1904.08405. 10.1109/TPAMI.2020.3008413 32750812

[B11] Gibson J. J. (1979). The Ecological Approach to Visual Perception. Boston, MA: Houghton Mifflin.

[B12] Hines M. Carnevale T. (2013). NEURON Simulation Environment. New York, NY: Springer.

[B13] Koenig N. Howard A. (2004). Design and use paradigms for gazebo, an open-source multi-robot simulator, in IEEE/RSJ International Conference on Intelligent Robots and Systems (Sendai), 2149–2154.

[B14] Menard M. (2011). Game Development with Unity, 1st Edn. Boston, MA: Course Technology Press.

[B15] Mitrokhin A. Sutor P. Fermüller C. Aloimonos Y. (2019). Learning sensorimotor control with neuromorphic sensors: toward hyperdimensional active perception. Sci. Robot. 4:eaaw6736. 10.1126/scirobotics.aaw6736 33137724

[B16] Rohmer E. Singh S. P. N. Freese M. (2013). V-rep: a versatile and scalable robot simulation framework, in Proceedings of the International Conference on Intelligent Robots and Systems (IROS).

[B17] Sanders A. (2016). An Introduction to Unreal Engine 4. Natick, MA: A. K. Peters, Ltd.

[B18] Shah S. Dey D. Lovett C. Kapoor A. (2017). Airsim: high-fidelity visual and physical simulation for autonomous vehicles, in Proceedings of the 11th Conference on Field and Service Robotics (FSR 2017) (Zurich).

[B19] Stimberg M. Brette R. Goodman D. F. (2019). Brian 2, an intuitive and efficient neural simulator. eLife 8:e47314. 10.7554/eLife.47314 31429824 PMC6786860

